# Glucocerebrosidase: Functions in and Beyond the Lysosome

**DOI:** 10.3390/jcm9030736

**Published:** 2020-03-09

**Authors:** Daphne E.C. Boer, Jeroen van Smeden, Joke A. Bouwstra, Johannes M.F.G Aerts

**Affiliations:** 1 Medical Biochemistry, Leiden Institute of Chemistry, Leiden University, Faculty of Science, 2333 CC Leiden, The Netherlands; d.e.c.boer@lic.leidenuniv.nl; 2 Division of BioTherapeutics, Leiden Academic Centre for Drug Research, Leiden University, Faculty of Science, 2333 CC Leiden, The Netherlands; jvansmeden@chdr.nl (J.v.S.); bouwstra@lacdr.leidenuniv.nl (J.A.B.); 3 Centre for Human Drug Research, 2333 CL Leiden, The Netherlands

**Keywords:** glucocerebrosidase, lysosome, glucosylceramide, skin, Gaucher disease

## Abstract

Glucocerebrosidase (GCase) is a retaining β-glucosidase with acid pH optimum metabolizing the glycosphingolipid glucosylceramide (GlcCer) to ceramide and glucose. Inherited deficiency of GCase causes the lysosomal storage disorder named Gaucher disease (GD). In GCase-deficient GD patients the accumulation of GlcCer in lysosomes of tissue macrophages is prominent. Based on the above, the key function of GCase as lysosomal hydrolase is well recognized, however it has become apparent that GCase fulfills in the human body at least one other key function beyond lysosomes. Crucially, GCase generates ceramides from GlcCer molecules in the outer part of the skin, a process essential for optimal skin barrier property and survival. This review covers the functions of GCase in and beyond lysosomes and also pays attention to the increasing insight in hitherto unexpected catalytic versatility of the enzyme.

## 1. Introduction

**Figure 1:**
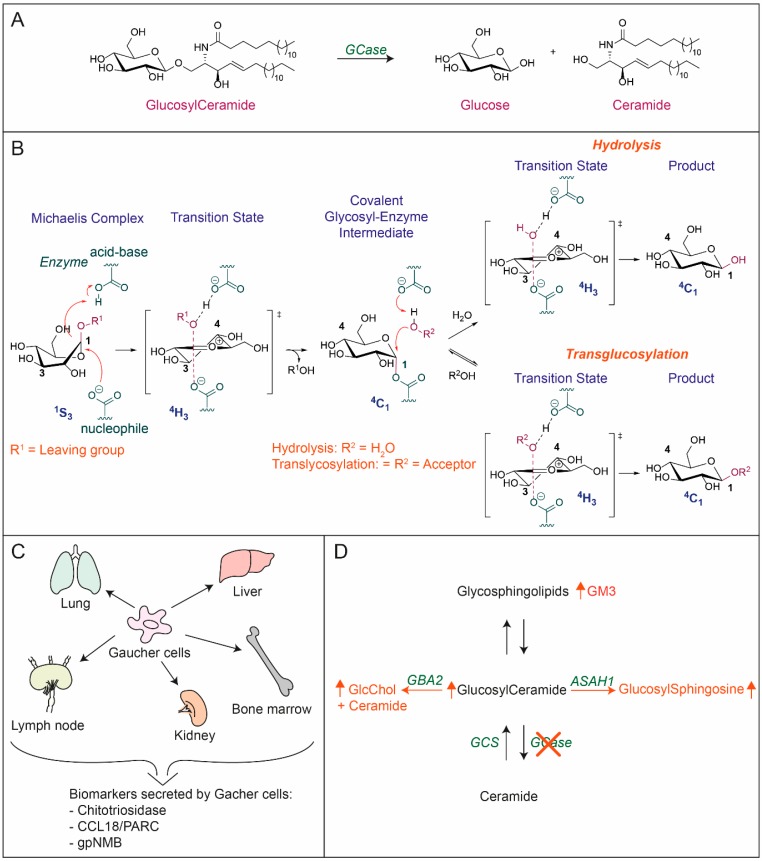
(**A**) Structure of glucosylceramide (GlcCer) and degradation by GCase to glucose and ceramide. (**B**) Catalytic activity GCase: Hydrolyzation of β-glucosides and transglucosylation activity. (**C**) Occurrence of Gaucher cells and the biomarkers they secrete in plasma. (**D**) Metabolic adaptations to GCase deficiency: increase of GlcCer as a result of lack of degradation by GCase. Accumulated GlcCer is converted by ASAH1 to glucosylsphingosine, Glucosylated cholesterol (GlcChol) formed by GBA2 increases, and GM3 levels rise because increased anabolism by glycosyltransferases to complex GSLs. Enzymes are depicted in green. ASAH1: acid ceramidase, GBA2: cytosolic β-glucosidase, GCase: β-glucocerebrosidase, GCS: glucosylceramide synthase.

The cellular acid β-glucosidase (EC 3.2.1.45) was first reported to be located in lysosomes more than 50 years ago [1]. There it degrades the glycosphingolipid glucosylceramide (GlcCer), also known as glucocerebroside (Figure 1A) [2]. The enzyme, commonly named glucocerebrosidase (GCase), is active towards GlcCer molecules with different fatty acyl moieties. Deficiency of GCase causes the recessively inherited disorder Gaucher disease (GD, OMIM #230800, ORPHA355), named after the French dermatologist Ernest Gaucher, who published the first case report [3]. A hallmark of GD are lipid-laden macrophages with lysosomal GlcCer deposits, referred to as Gaucher cells [4]. Numerous mutations in the *GBA* gene encoding GCase have been associated with GD [5]. The genetic heterogeneity contributes to the highly variable clinical manifestation of the disorder that may involve various organs and tissues [4]. A complete absence of GCase activity is incompatible with terrestrial life due to a disturbed skin barrier [6,7]. The lethal impairment stems from the crucial extracellular role of GCase in the stratum corneum (SC). This review covers the functions of GCase in the metabolism of GlcCer inside lysosomes and beyond. First, Section 2, Section 3, Section 4 and Section 5 deal with GCase as a cellular lysosomal enzyme, and in the second part Section 6 onwards focuses on the extracellular function of GCase in the skin.

## 2. Part 1: GCase and Lysosomal Glucosylceramide Degradation

### 2.1. Glucosylceramide as Intermediate of Glycosphingolipids

**Figure 2:**
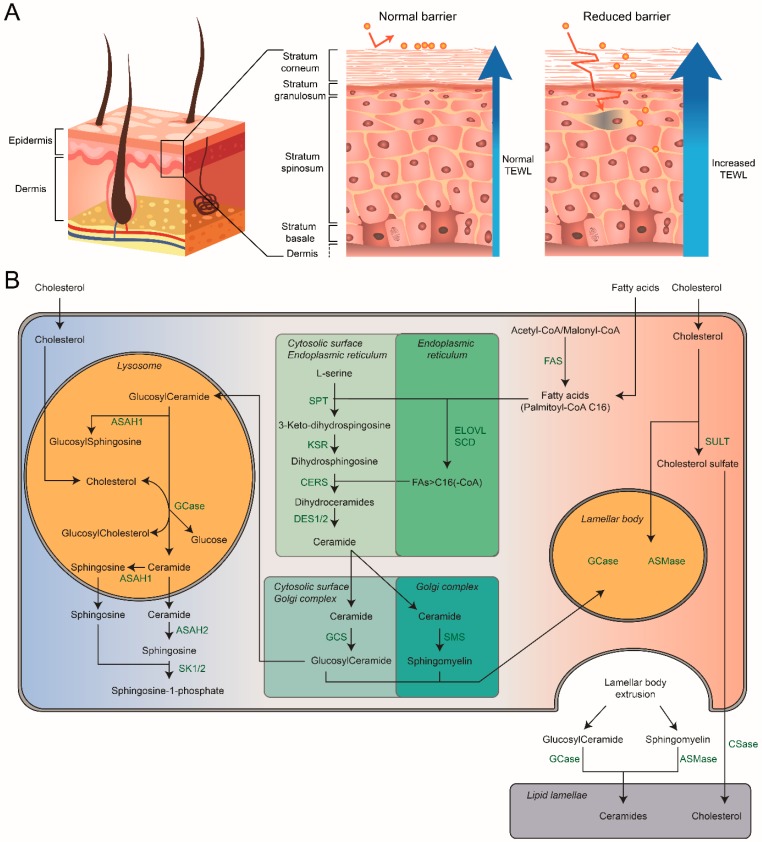
Schematic overview of the human skin and the main processes involved around GCase and its related lipids. (**A**) Schematic overview of a cross section of the skin showing the epidermis, dermis and subcutaneous tissue. The middle illustration shows a more detailed view of the epidermis under healthy conditions. The right illustration depicts a more detailed view of the epidermis with a reduced barrier. Exogenous compounds can get into deeper layers of the epidermis when the barrier is reduced, resulting in an immune response. It also leads to an increased transepidermal water loss (TEWL). (**B**) Schematic overview of the main processes involved around GCase within the cell. Arrows indicate the transport or conversion of lipids; associated enzymes are listed adjacent to their abbreviations. ASAH1: acid ceramidase, ASAH2: neutral ceramidase, ASMase: acid sphingomyelinase, CERS: ceramide synthase family, CSase: cholesterol sulfatase, DES1/2: dihydroceramide desaturase 1 and 2, ELOVL: elongation of very long chain fatty acids family, FAS: fatty acid synthase, GCase: β-glucocerebrosidase, GCS: glucosylceramide synthase, KSR: 3-ketosphinganine reductase, PLA-2: phospholipase, SCD: stearoyl-CoA desaturase, SMS: sphingomyelin synthase, SPT: serine palmitoyltransferase, SULT: cholesterol sulfotransferase type 2 isoform 1b.

The primary physiological substrate of GCase is GlcCer, the simplest glycosphingolipid (GSL) in which a single glucose β-glucosidic is linked to the 1-hydroxy of ceramide (Cer) [8]. Figure 2 presents an overview of the GSL metabolism. De novo formation of Cer starts on the endoplasmic reticulum (ER) with formation of 3-keto-dihydrosphingosine by the enzyme serine palmitoyl transferase (SPT) that conjugates the amino acid serine with a palmitoyl chain [9,10,11,12]. Next, the enzyme 3-ketosphinganine reductase (KSR) converts 3-keto-hydrosphingosine to dihydrosphingosine (sphinganine). Ceramide synthases (CERS) are responsible for acylation of dihydrosphingosine, thus generating diverse dihydroceramides [13,14,15]. In mammals six distinct CERS enzymes with different fatty acyl-CoA affinities have been identified. Subsequently, dihydroceramide desaturase (DES) catalyzes the conversion of dihydroceramides into ceramides ^15^. Ceramide is alternatively formed in the salvage pathway by acylation of sphingosine molecules released from lysosomes [16,17]. Cer can be further metabolized by conjugation of its 1-hydroxy, resulting in very diverse structures like ceramide 1-phosphate (C1P), sphingomyelin (SM), 1-O-acylceramide, galactosylceramide (GalCer), and GlcCer (reviewed in [18]). Formation of GlcCer, the key GSL of this review, involves transfer of Cer to the cytosolic surface of the Golgi apparatus where the membrane-bound glucosylceramide synthase (GCS) generates GlcCer using UDP-glucose as sugar donor and Cer as acceptor [19,20]. Next, some of the newly formed GlcCer molecules are converted back to Cer by the cytosol facing β-glucosidase GBA2 [21], but most reach via an unknown mechanism the luminal membrane of the Golgi apparatus. There, conversion to more complex GSLs like gangliosides and globosides occurs through stepwise addition of additional sugar and sulfate moieties (the biosynthesis and vast structural heterogeneity of GSL is excellently reviewed in [13,22]).

The major destination of newly formed GSLs is the outer leaflet of the plasma membrane. At the cell surface, GSLs fulfill a variety of important functions. GSLs interact with cholesterol molecules via hydrogen bonds and hydrophobic van der Waal’s forces and spontaneously form semi-ordered lipid microdomains, commonly referred to as lipid rafts [23,24]. Hydrophilic *cis*-interactions among GSL headgroups promote lateral associations with surrounding lipid and proteins. Residing in the GSL-enriched domains are proteins involved in interactions of cells with the exterior (extracellular space and other cells) and mediating the associated intracellular signaling processes [24,25,26]. The GSL composition of lipid rafts may exert modulating effects in the cell’s response to triggers. One example in this respect is the insulin receptor whose signaling is negatively influenced by neighboring gangliosides such as GM3 in lipid rafts [27,28,29]. Pharmacological reduction of GSLs results in improved glucose homeostasis in obese insulin-resistant rodents [30]. Similarly, the epidermal growth factor (EGF) receptor is influenced by the GSL composition of microdomains in which it resides [31]. GSLs at the cell surface also play direct roles in adhesion/recognition processes. For example, specific GSLs are involved in binding of pathogenic viruses, microorganisms, and bacterial toxins [32,33]. The topic was recently reviewed [34]. Glycosphingolipid-enriched lipid rafts essentially contribute to immunological functions as, for example, activation of T cells [35,36,37,38].

### 2.2. Lysosomal Turnover of Glycosphingolipid

GSLs may exit cells from the plasma membrane through incorporation in high-density lipoproteins [39,40]. However, most of the GSLs are internalized from the plasma membrane via endocytosis involving multi-vesicular bodies within late endosomes. Similarly, exogenous GSLs, such as constituents of lipoproteins or components of phagocytosed apoptotic cells, also reach lysosomes by endocytic processes. Upon the delivery of internalized material to lysosomes, fragmentation of GSL components takes place by step-wise removal of terminal sugars by specialized glycosidases. The process is further assisted by accessory proteins such as saposins A-D and GM2 activator protein (reviewed in [41]). The final lipid product of lysosomal fragmentation of GSLs, GalCer, and SM is in all cases Cer [41]. The lysosomal acid ceramidase (EC 3.5.1.23) subsequently splits Cer into free fatty acid and sphingosine to be exported to the cytosol [42]. The cytosolic sphingosine can then be used for the formation of Cer or can be converted by sphingosine kinases (SK1 and SK2) to sphingosine-1-phosphate (S1P) [43].

## 3. Glucocerebrosidase

### 3.1. GCase Protein and Life Cycle

The penultimate step in GSL degradation is the deglucosylation of GlcCer yielding glucose and Cer. This reaction is catalyzed by GCase, a 495 amino acid glycoprotein with four N-linked glycans [2,44]. GCase, based on its structural features, is classified in the glycoside hydrolase family GH30 (formerly in the related family GH5) [45]. The 3-D structure of GCase was resolved by crystallography [46,47]. GCase, like other GH5 and GH30 glycosidases, has an (α/β)_8_ TIM barrel catalytic domain. In the case of GCase this is fused with a β-structure consisting of an immunoglobulin-like fold [45]. GCase is a retaining β-glucosidase hydrolyzing a glucosidic substrate with net retention of glucose stereochemistry (Figure 1B).

Retaining beta-glucosidases generally use a two-step catalytic mechanism. The Koshland double displacement mechanism involves a catalytic nucleophile and acid/base residue [48]. A nucleophilic attack to the anomeric carbon of the glycosidic substrate is the first step. The aglycon is released assisted by a proton transfer from the acid/base residue and a covalent enzyme–glycoside complex is formed. Next, an activated water molecule deglycosylates the nucleophile, allowing a new round catalysis. The reaction involves two transient oxocarbenium ion-like states and the sugar substrate adopts different itineraries depending on its pyranose ring configuration [49]. In the case of retaining β-glucosidases like GCase, the substrate itinerary is ^1^S_3_ → ^4^H_3_ → ^4^C_1_ → ^4^H_3_ → ^4^C_1_ for the Michaelis complex → transition state → covalent intermediate → transition state → product [50,51]. In the (α/β)_8_ TIM barrel catalytic domain of GCase, E340 acts as nucleophile and E235 as acid/base residue [52,53].

Cyclophellitol, present in the mushroom *Phellinus* sp., is a potent irreversible inhibitor that binds covalently, in mechanism-based manner, to the nucleophile E340 of GCase [52,53,54]. The structurally related compounds cyclophellitol aziridine and conduritol B-epoxide inactivate GCase via the same mechanism [52,55]. Recently, superior suicide inhibitors for GCase were designed [56]. Cyclophellitol derivatives carrying a large hydrophobic substituent at C8 inactivate GCase with even higher affinity and with great specificity (not reacting with another retaining β-glucosidase like GBA2 and GBA3) [56,57]. Using cyclophellitol as scaffold, selective activity-based probes (ABPs) toward GCase were designed [52]. A reporter group (biotin or BODIPY) was attached to the C8 of cyclophellitol via a pentyl linker rendering ABPs allowing ultrasensitive and specific visualization of GCase in vitro and in vivo [58]. Subsequently, cyclophellitol aziridine ABPs with attached reporter groups via alkyl or acyl linkers were designed reacting with multiple retaining glycosidases in the same class [55,59]. Cyclophellitol aziridine ABPs labeling α-galactosidases, α-glucosidases, α-fucosidase, α-iduronidase, β-galactosidases, and β-glucuronidase, as well as cyclophellitol ABPs labelling galactocerebrosidase, were designed [60,61,62,63,64,65]. Applications of ABPs are the quantitative detection and localization of glycosidases in cells and tissues, as well as identification and characterization of glycosidase inhibitors by competitive ABP profiling [66,67].

GCase shows an acid pH optimum of hydrolytic activity, coinciding with the lysosomal pH [44]. The activity of the enzyme towards GlcCer is promoted by negatively charged lipids and saposin C, an activator protein generated in the lysosome by proteolytic processing of prosaposin [41,68]. The half-life of GCase in lysosomes is relatively short due to proteolytic degradation by cathepsins as suggested by the protective effect of leupeptin [69,70]. It was noted that unfolding and degradation of GCase is protected by occupation of the catalytic pocket [69].

GCase fundamentally differs from other lysosomal hydrolases in the mechanism underlying sorting and transport to lysosomes [44]. While most soluble lysosomal hydrolases are transported to lysosomes by mannose-6-phosphate receptors, this is not the case for GCase. In the inherited disorders mucolipidoses II and III, where formation of mannose-6-phosphate recognition signal in N-glycans of lysosomal hydrolases is impaired and consequently these enzymes are largely secreted, the transport of GCase to lysosomes is normal. In fact, in cultured skin fibroblasts the four N-glycans of GCase do not acquire mannose-6-phosphate [71]. Following correct folding of newly formed GCase molecules in the ER, these bind to the membrane protein LIMP2 (lysosomal membrane protein 2) [72,73,74]. This binding is mediated by hydrophobic helical interfaces on both proteins [75]. Action myoclonus renal failure syndrome (AMRF) is a recessively inherited disease caused by mutations in LIMP2 [76]. In most cell types of AMRF patients, except for phagocytic cells, GCase is markedly reduced due to faulty transport to lysosomes [76,77]. More recently, progranulin (PGRN) was identified as another factor influencing GCase [78,79]. PGRN is thought to function as a chaperone facilitating the transport of GCase to lysosomes. It recruits heat shock protein 70 (HSP70) to the GCase/LIMP2 complex in the ER and thus promotes delivery of GCase to lysosomes [80]. Another protein found to interact with newly formed GCase in the ER is ERdj3 [81].

### 3.2. Catalytic Activity of GCase

The primary substrate of GCase is GlcCer, as is reflected by the prominent accumulation of this lipid during GCase deficiency [82,83,84]. However, it recently has become apparent that catalytic versatility of the enzymes needs consideration. Firstly, GCase was found able to hydrolyze artificial β-xylosides [20]. Secondly, several retaining β-glycosidases are reported to be able to transglycosylate when provided with a suitable aglycon acceptor (Figure 1B) [85]. Such catalytic activity has also been observed for GCase, the enzyme being able to generate glucosylated cholesterol (GlcChol) by transglucosylation [86,87,88]. This reaction occurs during cholesterol accumulation in lysosomes as occurs in Niemann–Pick disease type C (NPC) [86]. Massive accumulation of GlcChol in the liver of NPC mice was demonstrated. Inducing lysosomal cholesterol accumulation in cultured cells by their exposure to U1986663A is accompanied by formation of GlcChol [86]. Of note, under normal conditions GlcChol is primarily degraded by GCase into glucose and cholesterol. It may be envisioned that further research will reveal that there exist more β-glucosidic metabolites being substrates (and products) of GCase.

## 4. Gaucher Disease, Inherited Deficiency in GCase

### 4.1. Gaucher Disease, a Lysosomal Storage Disorder

Since degradation of GSLs is catalyzed by lysosomal glycosidases, inherited deficiencies in these enzymes cause lysosomal accumulation of their GSL substrates, so-called glycosphingolipidoses [9,41,89,90,91]. Examples of such disorders are Gaucher disease, Krabbe disease, GM2-gangliosidosis, Sandhoff disease, and GM1-gangliosidosis. The glycosphingolipidoses are clinically diverse and generally show marked heterogeneity in severity of disease that usually involves neuropathy in more severely affected patients.

Gaucher disease is a prototype glycosphingolipidosis. The first case report was published in 1882 by Ernest Gaucher concerning a female patient with unexplained massive splenomegaly without leukemia [3]. Soon it was recognized that this patient represented a distinct disease entity that was subsequently referred to as Gaucher’s disease or Gaucher disease (GD). Following the demonstration of abnormal accumulation of GlcCer in GD patients as the result of deficient GCase activity, the *GBA* gene encoding the acid β-glucosidase was cloned and characterized [4]. The *GBA* gene is located at locus 1q21 and neighbored by a pseudogene [92]. Numerous mutations in *GBA* have now been associated with GD. The consequences of mutations at the level of the GCase protein may markedly differ. For example, the common N370S GCase mutation among Caucasians results in near normal production of a mutant enzyme with aberrant catalytic properties [93]. The heteroallelic presence of this mutation protects against a neuronopathic disease course [4,44,94]. This *GBA* mutation occurs relatively frequently among Ashkenazim and was proposed to offer some advantage against an infectious disease, for example, bubonic plaque or tuberculosis [95,96,97,98,99]. In contrast, another common pan-ethnic mutation encoding L444P GCase, results in a mutant enzyme that largely misfolds in the ER and consequently only a small fraction (<10% of normal) reaches lysosomes. Homozygosity for the L444P mutation is always associated with a severe neuronopathic disease manifestation [4]. The L444P mutation is thought to have arisen repeatedly by homologous recombination of the *GBA* gene with its pseudogene.

The genetic heterogeneity of GD is accompanied by clinical heterogeneity of the disorder. Common symptoms manifesting in GD patients are hepatosplenomegaly, hematological abnormalities like anemia and thrombocytopenia, skeletal disease, and neuropathology. A very severe manifestation (referred to as collodion baby) involves lethal skin barrier dysfunction [4]. Discrete phenotypic variants of GD are historically discerned: type 1, the non-neuronopathic variant; type 2, the acute neuronopathic variant; type 3, the subacute neuronopathic variant and the collodion baby or neonatal variant. It was proposed to no longer adhere to this classification, but rather view GD as a continuum of phenotypes [99]. Marked intraindividual variation occurs in type 1 GD patients in the nature and degree of organ involvement and particular symptoms such as skeletal disease [4].

The correlation of *GBA* genotype with GD phenotype is limited in some aspects. While the presence of N370S GCase protects GD patients against neuropathology, there are several reports of monozygotic GD twins with discordant severity of visceral disease [100,101]. A very specific clinical course is associated with the presence of D409H GCase involving yet unexplained cardiac symptoms, including aortic valve, mitral valve, and ascending aorta calcifications [102,103,104].

Modifier genes, and possibly epigenetics and external factors, are considered to impact on the clinical manifestation of GCase deficiency. The transmembrane protein CLN8 (ceroid-lipofuscinosis, neuronal 8), recycling between the ER and Golgi apparatus, is a putative modifier [105]. CLN8, identified as putative modifier of GD in a genome-wide association study, has recently been reported to be involved in the transport of newly formed lysosomal enzymes between ER and Golgi [106]. Other proteins are known to directly influence the life cycle and activity of GCase. Saposin C is the lysosomal activator protein of GCase, and patients with a defective saposin C develop symptoms similar to GD patients [68]. LIMP2, encoded by the *SCARB2* (scavenger receptor class B, 2) gene, transporting GCase to lysosomes was reported to be a GD modifier [107]. Polymorphisms in the *UGCG* gene coding for GCS catalyzing synthesis of GlcCer have also been proposed as GD modifiers [108]. Recently, microRNAs up- or downregulating GCase and downregulating LIMP2 were reported [109].

It has recently been appreciated that carrying a mutant *GBA* gene is not without health risk. Carriers of GD have a yet unexplained significantly increased (20-fold) risk for developing Parkinson disease (PD) and Lewy body dementia (LBD) [110,111,112]. A recent study in the United Kingdom revealed that 5–25% of patients with PD carry glucocerebrosidase gene mutations, and 10–30% of glucocerebrosidase carriers will develop PD by age 80 [113]. Of note, active GCase activity is also decreased, and corresponding glycosphingolipid substrate levels elevated, in the brain in PD without GBA1 mutations [114,115]. Abnormalities in multiple enzymes and other proteins involved in sphingolipid metabolism were observed in association with PD [114,116,117]. With increasing age, the brain of mice shows reduced GCase levels and increased amounts of lipid substrate [115]. PD is historically viewed as a “proteinopathy” with cellular protein aggregates like that of α-synuclein (αSyn). It has more recently been hypothesized that sphingolipid abnormalities may be primary disturbances that can produce protein aggregation [114]. Indeed, inactivation of GCase promotes accumulation of αSyn aggregates [118]. It was observed that insoluble alpha-synuclein positive aggregates in sporadic PD midbrain linearly correlate with loss of GCase activity [119]. Likewise, protein aggregates develop in mice with primary GBA mutations [120]. Supplementation of GCase or reduction of accumulating glycolipids prevents and reverses α-synucleinopathy [121,122]. It was furthermore observed that over-expression of aggregating αSyn causes a reduction of GCase, suggesting a potential harmful interaction between the two proteins in a self-amplifying manner [123,124,125]. In vitro experiments showed that GCase and aSyn may directly interact at lysosomal pH [126]. Different explanations have been proposed for ways by which mutant GCase may induce α-synucleinopathy (reviewed in [125,127,128]). For example, it was hypothesized that the accumulation of substrates of GCase is pathogenic; that GCase deficiency causes inhibition of autophagy and lysosomal degradative capacity and subsequently reduces turnover of αSyn; that increased αSyn levels impair the activity of GCase and vice versa; and, that GCase deficiency impairs mitochondria. Contrarily, it was proposed that mutant GCase protein may be toxic by inducing an excessive unfolded protein response in the ER or saturating the ubiquitin–proteasome pathway [129,130]. It is conceivable that multiple mechanisms may be involved in the GBA-PD pathology.

### 4.2. Lysosomal GlcCer Deposits in Macrophages: Gaucher Cells

The storage of GlcCer in GD patients occurs almost exclusively in macrophages residing in the spleen, liver, bone marrow, lymph nodes, and lung (Figure 1C) [131]. The lipid-laden Gaucher cells are viable, alternatively activated macrophages [132]. These cells overproduce and secrete specific proteins resulting in massively elevated plasma levels in symptomatic GD patients. These proteins are now used as biomarkers of body burden of Gaucher cells. The first identified plasma biomarker is the chitinase named chitotriosidase encoded by the *CHIT1* gene [133,134]. It can be conveniently detected by the measurement of its activity towards 4-methylumbelliferyl-chitotrioside [133] and the superior substrate 4-metylumbelliferyl-4′-deoxy-chitobioside [135,136]. Plasma chitotriosidase is on average about 1000-fold elevated in type 1 GD patients. Immunohistochemistry and in situ hybridization revealed that the enzyme is produced by Gaucher cells. Common is a 24-base-pair duplication in the *CHIT1* gene that excludes synthesis of active chitinase [137]. The chemokine CCL18/PARC (Chemokine (C-C motif) ligand 18; Pulmonary and activation-regulated chemokine) serves as an alternative plasma marker of Gaucher cells, being 20 to 50-fold elevated plasma of type 1 GD patients [138,139]. The chemokine is over-produced and secreted by Gaucher cells [139]. More recently the glycoprotein nonmetastatic melanoma protein B (gpNMB) was found to be overproduced by Gaucher cells [140]. A soluble fragment of gpNMB is released into plasma and is over 50-fold elevated in type 1 GD patients [140,141]. In cerebral spine fluid and brain of type 3 GD patients elevated gpNMB levels have also been observed [142]. Likewise, recently an increased level of gpNMB in the substantia nigra of PD patients was reported [143]. In mice with conditional deficiency in GCase in the white blood cell lineage Gaucher-like cells are formed. These do not produce chitotriosidase or CCL18, but gpNMB does [140,144]. Inactivation of GCase with an irreversible inhibitor was found to increase gpNMB in the brain [143]. Interestingly, zebrafish and fruit flies overproduce a chitinase during GCase deficiency [130,145].

There is compelling evidence for a direct role of Gaucher cells in GD pathology. Their presence in spleen, liver, and bone marrow is associated with splenomegaly, hepatomegaly, and hematological abnormalities, respectively [4]. The same holds for these symptoms in GD mice with induced GCase deficiency in white blood cells [144]. In GD spleens the storage lesions contain a core of mature Gaucher cells surrounded by pro-inflammatory macrophages [132]. These lesions likely contribute to the complex cytokine, chemokine, and protease abnormalities in GD patients [91,146,147]. Type 1 GD patients show low-grade inflammation and activation of both coagulation and the complement cascade [148,149]. Of note, many of the visceral symptoms of type 1 Gaucher disease patients resemble those of Niemann–Pick type A and B patients suffering from lysosomal acid sphingomyelinase (ASMase) deficiency causing lysosomal sphingomyelin storage [42]. In both disorders, lipid storage in visceral macrophages is a hallmark. In sharp contrast, while GCase is markedly reduced in most cell types of LIMP2-deficient AMRF patients, their symptoms differ from those of type 1 Gaucher patients. Likely this is due to the fact that macrophages of AMRF patients contain a high residual GCase and consequently no lipid-laden macrophages are formed [77].

### 4.3. Therapies of Gaucher Disease: ERT, SRT, PCT/EET

The prominence of lipid-laden macrophages in GD and their relationship to pathology has prompted the design of rational therapies aiming to prevent and/or correct the lipid-laden macrophages. The first effective treatment designed for type 1 GD is enzyme replacement therapy (ERT) aiming to supplement patient macrophages with lacking enzyme by repeated intravenous enzyme infusion [150]. Therapeutic GCase, nowadays recombinant but initially isolated from placenta, has enzymatically modified N-linked glycans with terminal mannose groups to favor uptake via the mannose receptor (or another mannose-binding lectin) at the surface of tissue macrophages. Two weekly ERTs reverse hepatosplenomegaly and hematological abnormalities in type 1 GD patients [67]. In addition, it reduces storage cells in the bone marrow [151]. Present ERT does, however, not prevent neurological symptoms due to the inability of enzyme to pass the blood brain barrier.

An alternative GD treatment is substrate reduction therapy (SRT) [152,153,154]. SRT aims to balance synthesis of GlcCer with reduced GCase activity of GD patients. Oral inhibitors of GCS (Miglustat and Eliglustat) are approved drugs. Eliglustat therapy resembles ERT in efficacy [155]. Brain-permeable inhibitors of GCS are presently designed and tested [156]. The response to treatment of GD patients is primarily monitored by clinical assessments. A retrospective evaluation revealed that reductions in plasma chitotriosidase during ERT correlate with corrections in liver and spleen volumes, improvements in hemoglobin, platelet count, and bone marrow composition [157]. Given the observed positive outcome of bone marrow transplantation in type 1 GD patients, genetic modification of hematopoietic stem cells was, and still is, seriously considered as therapeutic avenue [144].

At present there is still an unmet need for neuronopathic GD. Small compounds are actively studied as potential therapeutic agents in this respect. One envisioned approach is pharmacological chaperone therapy (PCT). Chemical chaperones are small compounds improving folding of mutant GCase in the ER, thus increasing lysosomal enzyme levels. Current studies with ambroxol, a weak inhibitor of GCase, indicate impressive reductions in spleen and liver volumes in ambroxol-treated type 1 GD patients as well as clinical improvements in type 3 GD patients [158,159,160]. Another approach is enzyme enhancement therapy with small compounds (EET). An example of this is arimoclomol, a heat shock protein amplifier, found to improve refolding, maturation, and lysosomal activity of GCase in GD fibroblasts and neuronal cells [161].

## 5. Metabolic Adaptations to Lysosomal GCase Deficiency

### 5.1. Formation of Glucosylsphingosine From Accumulating GlcCer

Important metabolic adaptations occur during GCase deficiency in lysosomes (Figure 1D) [162]. We demonstrated that part of the accumulating GlcCer is actively converted by lysosomal acid ceramidase to glucosylsphingosine (GlcSph) [163]. GlcSph is sometimes also referred to as lyso-GL1 or lyso-GB1. It was earlier observed that GlcSph is increased in the brain and spleen of GD patients [164,165]. We firstly reported an average 200-fold increased GlcSph level in plasma of symptomatic type 1 GD patients [166]. Urine of GD patients also contains increased GlcSph isoforms [167]. Pharmacological inhibition of GCase in cultured cells and zebrafish embryos causes a rapid increase in GlcSph [168]. The quantitative detection of GlcSph in biological samples was improved by o-phthaldialdehyde (OPA) derivatization and high-performance liquid chromatography [169]. Further improvement was reached by the introduction of LC-MS/MS (liquid chromatography-mass spectrometry) employing an identical (13)C-encoded glucosylsphingosine standard [168]. Measurement of elevated plasma GlcSph is now regularly used in the confirmation of GD diagnosis.

Excessive GlcSph in GD patients is believed to contribute to various symptoms. GlcSph was linked to the common reduced bone mineral density (osteopenia) in GD patients by impairing osteoblasts [170]. It is reported to promote α-synuclein aggregation, a hallmark of Parkinson disease [171]. Antigenicity of GlcCer, and possibly GlcSph, is thought to cause the common gammopathies in GD patients, gammopathies that can lead to multiple myeloma [172]. The same lipids were proposed to activate the complement cascade activation and associated local tissue inflammation [173]. GlcSph is hypothesized to diminished cerebral microvascular density in mice, based on the observed interference of the lipid with endothelial cytokinesis [174]. Earlier studies have provided evidence that GlcSph promotes lysis of red blood cells, impairs cell fission during cytokinesis, damages specific neurons, interferes with growth, and activates pro-inflammatory phospholipase A2 (see for a review [91]). In line with these observations is the occurrence of hemolysis, multinucleated macrophages, neuropathology, growth retardation, and chronic low-grade inflammation in GD patients [4]. Of note, in the brain of ageing mice reduction of active GCase in combination with increased glucosylceramide and glucosylsphingosine levels were observed [116].

The conversion of accumulating GSL in lysosomes to glycosphingoid bases (lyso-lipids) is not unique to Gaucher disease. Comparable acid ceramidase-dependent formation of sphingoid bases occurs in Krabbe disease (galactosylsphingosine), Fabry disease (globotriaosylsphingosine; lysoGb3), GM2-gangliosidosis (lysoGM1), and GM2-gangliosidoses (lysoGM2) [91,175]. In Niemann–Pick disease types A and B, the water soluble lysoSM is formed from accumulating SM [176]. As for GlcSph in GD, toxicity of excessive galactosylsphingosine in Krabbe disease and excessive lysoGb3 in Fabry disease have been proposed [91,177,178,179,180,181,182].

### 5.2. Excessive Gangliosides

In GD patients increases of the ganglioside GM3 (monosialodihexosylganglioside) in plasma and spleen were observed [183]. It is unknown whether this abnormality is caused by increased metabolic shuttling of newly formed GlcCer to gangliosides and/or impaired recycling of gangliosides. Not surprisingly (see Section 2.1), the elevated concentrations of GM3 in GD patients are accompanied by insulin insensitivity, without overt hyperglycemia [184].

### 5.3. Increased Activity of Cytosol-Faced GBA2 and GlcChol

Besides GCase, cells contain another retaining β-glucosidase that metabolizes GlcCer. The enzyme GBA2 was discovered during studies with GCase-deficient cells [21]. GBA2 is synthesized as soluble cytosolic protein that rapidly associates to the cytosolic leaflet membranes with its catalytic pocket inserted in the lipid layer. GBA2 shows prominent transglucosylase capacity and is largely responsible for the (reversible) formation of GlcChol from GlcCer and cholesterol [86]. The *GBA2* gene (locus 1p13) was identified and GBA2-deficient mice have meanwhile been generated [185,186]. The animals develop normally without overt abnormality, except for incidences of male infertility [185]. GBA2-deficient zebrafish also develop normally [168]. Inhibition of GBA2 in GD and NPC patients treated with N-butyldeoxynojirimycin causes no major complications, whereas on the other hand, individuals with spastic paraplegia and cerebellar ataxia were found to be GBA2 deficient [187,188,189,190]. The physiological role of the highly conserved GBA2 is still an enigma [191].

Reducing GBA2 activity, genetically or using small compound inhibitors such AMP-DNM, has remarkable beneficial effects in NPC mice, ameliorating neuropathology and prolonging lifespan significantly [52,53]. A comparable neuro-protective effect of the iminosugar AMP-DNM was also observed in mice with Sandhoff disease, another neuropathic glycosphingolipidosis [54]. Presently zebrafish models are used to study the poorly understood interplay between GCase and GBA2-mediated metabolism of GlcCer [168]. The possible toxic effect of excessive glucosylated metabolites generated by GBA2 during GCase deficiency warrants further investigation.

## 6. Part 2: GCase and Glucosylceramide Metabolism Beyond the Lysosome

### 6.1. GCase: Other Locations Than Lysosomes

As discussed in Section 3.1, GCase does not rely on mannose-6-phosphate receptor-mediated intracellular sorting and re-uptake after secretion. The intracellular transport of GCase is tightly governed by the membrane protein LIMP2 and secretion of GCase into the extracellular space is normally prevented [77]. Immuno-electron microscopy has revealed that specific organelles are involved in trafficking of GCase-LIMP2 complexes from the Golgi apparatus to lysosomes [192]. The delivery of GCase to other locations than lysosomes warrants consideration and discussion.

### 6.2. Lysosome-Related Organelles

To fulfill specific physiological functions several cell types have adapted their endolysosomal apparatus and evolved specialized secretory compartments, the lysosome-related organelles (LROs) (for reviews see [193,194]). The LROs are diverse and comprise endothelial cell Weibel-Palade bodies, cytotoxic T cell lytic granules pigment cell melanosomes, and platelet dense and alpha granules. Common components of LROs are tetraspanin CD63, and GTPases RAB27A or RAB27B. The same proteins also occur in multivesicular endosomes (MVEs) that excrete intraluminal vesicles (ILVs) as exosomes upon fusion with the plasma membrane [195]. The notochord vacuole in the zebrafish is also considered to be an LRO [196,197]. Interestingly, LIMP2, the GCase transporter protein, was implicated in the formation of this LRO [198].

An established link between GSLs and LROs concerns the pigmented melanosomes in melanocytes. The formation of melanosomes requires GSLs: melanoma cells when deficient in GCS lose pigmentation due to aberrant transport of the enzyme tyrosinase synthesizing melanin [199]. Similarly, cultured melanocytes lose pigmentation when treated with a GCS inhibitor (Smit and Aerts, unpublished observations).

Keratinocytes contain a special kind of LRO, the lamellar body (LB), which justifies more detailed discussion regarding GSLs and their metabolism (see Section 8 and Section 9). Prior to this, the composition of the mammalian skin is introduced in the section below.

## 7. Composition of the Skin

### 7.1. Skin Differentiation and Barrier Formation

The mammalian skin acts as a key barrier offering protection against xenobiotics and harmful pathogens and preventing excessive water loss from the body (Figure 2A) [200]. The barrier function resides in the epidermis, the outermost part of the skin that consists of four distinct layers: the stratum basale (SB), stratum spinosum (SS), stratum granulosum (SG), and stratum corneum (SC) [201]. The innermost SB, SS, and SG are the vital parts of the epidermis (thickness: 50–100 μm) while the SC is the non-vital differentiation product (thickness: 10–20 μm). The SB contains proliferating keratinocytes that after escape from this single cell layer start to differentiate and migrate towards the SC, where the keratinocytes differentiate to terminal corneocytes. During this differentiation process the keratinocytes flatten and diminish their water content. During the flattening process cells become filled with keratin. At the interface between the SG and SC, subcellular structures like organelles and nuclei are degraded and corneocytes are formed (as reviewed in [202]).

### 7.2. Stratum Corneum: Hydration and Skin-pH

Proper function and features of the SC are dependent on optimal water content and acidity. The SC hydration level depends on multiple factors such as amino acids, specific sugars and salts, referred to as the natural moisturizing factor (NMF) [203]. Amino acids of the NMF are breakdown products of the major SC protein filaggrin. Mutations in the filaggrin gene *FLG* cause a reduced NMF level associated with dry skin [204,205]. NMF also plays a key role in maintenance of pH in the SC. At the outside of the SC the pH is 4.5–5.3 and it gradually increases to pH 6.8 in the inner SC [206]. The local pH likely modulates the activity of various enzymes in the SC, including GCase and ASMase, with optimal catalytic activity at a more acid pH, and thus also impacts on lipid structures [207].

### 7.3. Stratum Corneum: Composition

The SC has a “brick-and-mortar” like structure, where the corneocytes are the “bricks” embedded in a lipid matrix that is the “mortar” of the SC [206,208]. During the terminal differentiation of corneocytes, plasma membranes develop into the cornified lipid envelope, a lipid-linked crosslinked protein structure [209]. The cornified lipid envelope acts as template for the formation and organization of extracellular lipid lamellae [210,211]. The lipid matrix contains approximately on a total lipid mass basis 50% ceramides, 25% cholesterol, and 15% free fatty acids with very little phospholipid. The adequate balance of lipid components is essential for proper lipid organization and SC barrier competence [212]. Alterations in the lipid composition have been associated to various skin diseases, particularly to psoriasis, atopic dermatitis and several forms of ichthyosis [213,214,215,216,217,218].

## 8. Sphingolipids of the Stratum Corneum

### 8.1. Role of Lamellar Bodies

Keratinocytes having specific ovoid-shaped LROs with a diameter of about 200 nm are called lamellar bodies (LBs), or alternatively lamellar granules, membrane-coating granules, cementsomes, or Odland bodies [219]. LBs have a bounding membrane surrounding lipid disks. The main lipids packed in LBs are precursors of ceramides and fatty acids constituting the lamellar matrix in the SC. In the uppermost granular cells, the bounding membrane of the LB fuses into the cell plasma membrane, and the lipid disks are extruded into the intercellular space between the SC and SG. The initially extruded content of the LB is largely metabolized to ceramides and fatty acids and rearranged to form together with cholesterol the intercellular lamellae of the SC.

Keratinocytes serve as the initial factory of the permeability barrier of the skin [219]. Briefly, the generation of SC barrier lipids initiates in keratinocytes, where ceramides are de novo formed by ceramide synthase 3 (CerS3). The sphingolipid content of keratinocytes increases along with differentiation. Newly formed ceramides are rapidly modified into glucosylceramides (GlcCers) and sphingomyelins (SMs), thereby likely protecting keratinocytes from cytotoxic ceramide effects. Next, these sphingolipids are packaged into LBs [212]. The membrane protein ABCA12 (ATP-binding cassette sub-family A member 12) is essential for the presence of GlcCer in LBs [220,221,222]. Several mutations in the *ABCA12* gene cause Harlequin-type ichthyosis, characterized by thickened skin over nearly the entire body at birth and causing early death. Incorporated in LBs besides lipids are also acid hydrolases including GCase, ASMase, and phospholipase A as well as proteases and antimicrobial peptides. Following exocytotic secretion of LBs, the SM and GlcCer molecules are largely enzymatically re-converted to ceramides [223,224].

### 8.2. Chemical Composition of Skin Sphingolipids

The sphingolipids in the skin differ in their complexity of chemical composition from those encountered in most tissues. Firstly, their sphingosine backbones are modified to yield from dihydroceramide (DS) precursors not only the regular ceramide (S) but 6-hydroxyceramide (H), phytoceramide (P) and 4,X-dihydroxysphinganine containing ceramide (T), as well [225,226,227,228]. In addition, skin ceramides have unique fatty acyl moieties. Besides regular non-hydroxylated fatty acyls of variable chain length, there are α-hydroxylated and ω-esterified structures (acylceramides) [229].

In keratinocytes, fatty acids can be elongated by elongases (mainly ELVOL1, ELVOL4, and ELVOL6) [230,231]. Very long chain fatty acids are incorporated in phospholipids and sphingolipids are packaged in LBs. Cholesterol does not require a conversion to be transported into LBs. Cholesterol can furthermore be metabolized to oxysterol or cholesterol sulfate. Oxysterol and cholesterol sulfate can both stimulate keratinocyte differentiation, additionally, cholesterol sulfate has a key role in [232,233,234,235]. Since cholesterol sulfate is highly amphiphilic it can cross the cell membrane and directly enter the SC, where it is metabolized by LB-derived steroid sulfatase to cholesterol [236,237]. Because cholesterol sulfate inhibits proteases that are involved in desquamation [238], its decrease in the upper layers of the SC results in the initiation of desquamation [239,240].

Besides the presence of regular ceramides, the scaffold of the lipid matrix in the SC is built of acylceramides, containing ω-hydroxylated very long chain fatty acids acylated at the ω-position with linoleic acid [212,228]. Also, the acylceramides are synthetized in the keratinocytes, where they and regular ceramides are glucosylated at Golgi membranes and secreted via LB secretion. Extracellularly the linoleic acid residues are replaced by glutamate residues at proteins exposed on the surface of corneocytes, thus completing the corneocyte lipid envelope [212,228,241].

## 9. GCase: Crucial Extracellular Role in the Skin

**Figure 3:**
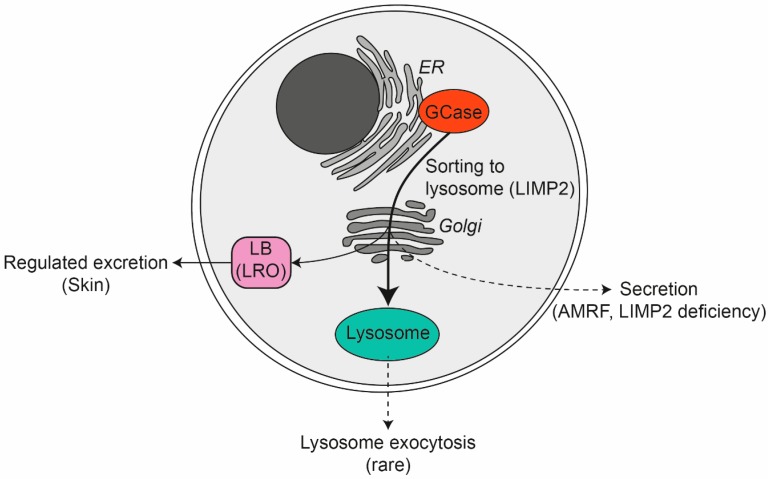
The life cycle of GCase in and beyond the lysosome. AMRF: Action myoclonus renal failure syndrome, ER: endoplasmic reticulum, LB: lamellar body, LIMP2: lysosomal membrane protein 2LRO: lysosome related organelle.

Inhibition of either cholesterol, phospholipid, ceramide or glucosylceramide synthesis prevents the delivery of lipids into LBs, disrupting LB formation, thereby impairing barrier homeostasis (Figure 3) [242]. LB secretion and lipid structure is abnormal in the outer epidermis of multiple skin diseases, like Atopic Dermatitis and Netherton syndrome [215,243,244]. A complete lack of GCase results in a disease phenotype (collodion baby) with fatal skin abnormalities and inhibition of GCase activity reduces the permeability barrier formation [245,246,247,248]. Gaucher mice homozygous for a null allele develop skin abnormalities that are lethal within the first day of life [6,7]. Holleran and colleagues showed increased trans-epidermal water loss (TEWL) and altered barrier function in GCase-deficient mice [248], suggesting deficient conversion of GlcCer to ceramides by GCase alters the skin barrier function. Identical changes were observed in hairless mice treated with GCase inhibitor bromoconduritol B epoxide, however, ceramide levels remained normal [246,248]. Similarly, mice deficient for prosaposin, and therefore also lacking the GBA activator protein saposin C, accumulate GlcCer in the SC and show abnormal SC lamellar membrane structures [249]. Interestingly, deficiency of LIMP2 in AMRF patients is not associated with skin abnormalities. No prominent abnormalities have also been noted in LIMP2-deficient mice. Apparently, GCase is reaching the SC sufficiently without its regular transporting protein.

GlcCer and GBA appear to be co-localized in the LB [250,251,252]. GBA activity has been observed throughout the outer parts of the epidermis [253,254,255], and recently a novel in situ method with the use of activity-based probes (ABPs) confirmed predominant localization of active GBA in the extracellular space of the SC lipid matrix [256].

GD is not the only lysosomal storage disease associated with skin barrier abnormalities. In Niemann–Pick disease a deficiency in ASMase causes an impaired conversion of SM into ceramides in the SC and, therefore, into a disturbed skin barrier [248,257]. Reduction of epidermal ASMase activity by the inhibitor imipramine causes delayed permeability barrier repair after SC injury [258].

## 10. Atopic Dermatitis

A common skin disease is atopic dermatitis (AD, OMIM #603165). Clinical manifestation of AD involves eczematous lesions as well as erythema, xerosis, and pruritis [259,260,261]. In AD there is a complex interplay between inflammation, genetic background, and the skin barrier. Inflammation can affect the skin barrier, and subsequent entry of compounds promotes an immune response. Additionally, it was observed that AD is associated with loss of function mutations in the filaggrin gene *FLG* [262,263]. As discussed in Section 7.2, filaggrin is essential for SC hydration and may affect the sensitivity of the skin [264]. Even though *FLG* mutations have been suggested as a predisposing factor for AD, they do not influence SC ceramide synthesis [264,265,266].

### 10.1. SC Lipids in AD

SC lipid metabolism and composition have been substantially studied in AD, however there is some disagreement in literature about the lipid composition in the skin of AD patients. Farwanah and co-workers reported no change in non-lesional AD skin compared to control [267], although other studies report a decrease in total ceramide level, as well as an increase in ceramide (AS) and a decrease in ceramide (EOS) and (EOH), mainly in lesional AD skin compared to control [215,266,268,269,270,271]. Additionally, Di Nardo et al. have reported a decrease in ceramide/cholesterol ratio in AD skin [271].

Besides subclass composition ceramide chain length has also been studied in AD. Some report an increase of short chain ceramides (total chain length of 34 carbon atoms) in lesional AD skin that also correlated with an increased TEWL [268,272]. Moreover, levels of ω-O-acyl-ceramides correlated negatively with TEWL [268]. A reduction in ω-O-acyl-ceramide in AD compared to control was also reported by Jungersted et al. They additionally observed no statistical difference between their *FLG* mutant and wild-type group in relation to the ω-O-acyl-ceramide decrease [266].

Data on fatty acids in relation to AD skin are limited, but there are a few reports reporting a reduced fatty acid chain length [273,274]. A study by van Smeden et al. described an increase of shorter fatty acids, mainly saturated fatty acids with 16 and 18 carbon atoms, as well as a reduction in fatty acids with 24 carbons or more in non-lesional AD patients [273]. However, another study observed an increased level of very long fatty acid chains in non-lesional as well as lesional AD [274]. It was hypothesized that SC ceramides and fatty acids share a common synthetic pathway, and this is consistent with the observation that ceramide composition is paralleled by the chain length of fatty acids [275].

The expression of enzymes involved in the biosynthesis of fatty acids and ceramides was related to the SC lipid composition in lesional AD skin [276]. Danso et al. observed an altered expression of GBA, ASMase, and CerS3 in lesional AD skin with a corresponding increase in ceramide (AS) and (NS) and decrease in esterified ω-hydroxy CERs. Additionally, they noted increased levels of unsaturated fatty acids and reduced levels of C22–C28 fatty acids in combination with an altered expression of stearoyl CoA desaturase (SCD) and elongase 1 (ELOVL1) [276].

### 10.2. Potential Role for Glucosylsphingosine in AD Pathology

Deficiency of ceramides in the SC is thought to contribute to the dry and barrier-disrupted skin of patients with AD. It was proposed that this deficiency involves a tentative novel enzyme named sphingomyelin-glucosylceramide deacylase, forming sphingosylphosphorylcholine (SPC; lysoSM) and GlcSph from SM and GlcCer. Increased deacylase activity is thought to contribute to reduced formation and subsequent deficiency of ceramide in the AD skin [277]. The deacylase enzyme is considered to be distinct from acid ceramidase as based by apparent isoelectric point [278]. Increased deacylase activity was observed for involved SC and epidermis in patients with AD [279]. Unfortunately, the deacylase has so far not been isolated and characterized. At present it cannot be excluded that the intriguing observations are explained by some neutral ceramidase, a bacterial amidase, or even acid ceramidase that in lipid-laden macrophages of GD patients shows GlcCer deacylase activity.

A common symptom in AD is pruritis. It was observed that GlcSph induces scratching in mice and more recently it was demonstrated that GlcSph activates the Serotonin Receptor 2 a and b, considered to be part of a novel itch signaling pathway [280,281].

### 10.3. Direct Role of GCase in AD?

As discussed above, GCase expression was found to be altered in (particularly lesional) AD skin [276]. However, no abnormality in GCase activity level in AD skin was previously noted [282]. Earlier research in mice pointed to changes in location of GCase activity in mice with a skin barrier disruption [246]. Using the specific and sensitive ABP technology, the localization of active GCase molecules in AD skin has been studied. An abnormal GCase localization in (mainly lesional) AD skin was observed together with abnormal SC lipids (Boer, submitted for publication). It will be of interest to comparably study other skin diseases. It should be stressed that abnormalities in GCase are not a sole cause for AD, however, an acquired local abnormal enzyme activity might contribute to the pathology.

## 11. Summary and Conclusions

This review addresses the multiple functions of the enzyme GCase that degrades the ubiquitous glycosphingolipid GlcCer. In the first part of the review, the metabolism and various functions of glycosphingolipids in health and disease are discussed. The structural features and catalytic mechanism of GCase are described, as well as its remarkable life cycle involving LIMP2-mediated transport to lysosomes. The essential cellular role of GCase in turnover of GlcCer in lysosomes is illustrated by the lysosomal storage disorder Gaucher disease (GD), which results from an inherited GCase deficiency. The review describes the variable symptoms of GD patients and the presumed underlying pathophysiological mechanisms. In addition, it addresses the presently available treatments of visceral manifestations of GD. In the second part of the review, attention is focused on another, extracellular, role of GCase in the skin. In the stratum corneum, GCase converts secreted GlcCer to ceramide, an essential component of lipid lamellae contributing to the barrier properties of the skin. A major lack of GCase activity causes a lethal skin pathology, the collodion baby.

To conclude, the catalytic ability of the enzyme GCase has been exploited in evolution for two different functions: in lysosomes, it essentially contributes to cellular glycosphingolipid metabolism, and in the extracellular space of the stratum corneum, it generates an essential building block for lipid lamellae.
